# Supplementary material for: Resolution of complex fluorescence spectra of lipids and nicotinic acetylcholine receptor by multivariate analysis reveals protein-mediated effects on the receptor's immediate lipid microenvironment
Source: PMC Biophys. 2008 Dec 18;1:6. doi: 10.1186/1757-5036-1-6 (PMC2666634; doi:10.1186/1757-5036-1-6)

REFERENCE SPECTRA OF THE PROBES

Figure 1. Examples of fluorescence emission spectra of AChR, PyPC and NBD-Chol. Total lipid concentration and lipid to protein molar ratio (in the AChR-containing liposomes) were 40 µM and 500:1, respectively. Temperature = 25℃.

Conditions of spectra.

1 - AChR (0.08 µM; Ex. 290 nm) in DOPA/DOPC/NBD-Chol (20:40:40).

2 - PyPC (2 µM; Ex. 340 nm) in pure DOPC. The almost imperceptible excimer band is due to the homogeneous distribution of the probe in this lipid fluid system (transition temperature: -20℃). Thus, the "apparent" concentra tion of the probe is low.

3 - PyPC (2 µM; Ex. 340 nm) in pure DPPC. The excimer band is more evident due to the formation of probe-enriched domains laterally segregated from the gel phase of DPPC (transition temperature: 41℃). There is an au gmented "apparent" concentration of the probe reported by the excimer formation.

4 - PyPC (2 µM; Ex. 290 nm) in DOPA/DOPC/NBD-Chol (30:30:40), showing the sum of the PyPC excimer and NBD-Chol emission bands (by FRET), beyond ~ 434 nm.

5 - NBD-Chol (16 µM; Ex. 478 nm) in DOPA/DOPC/NBD-Chol (30:30:40).

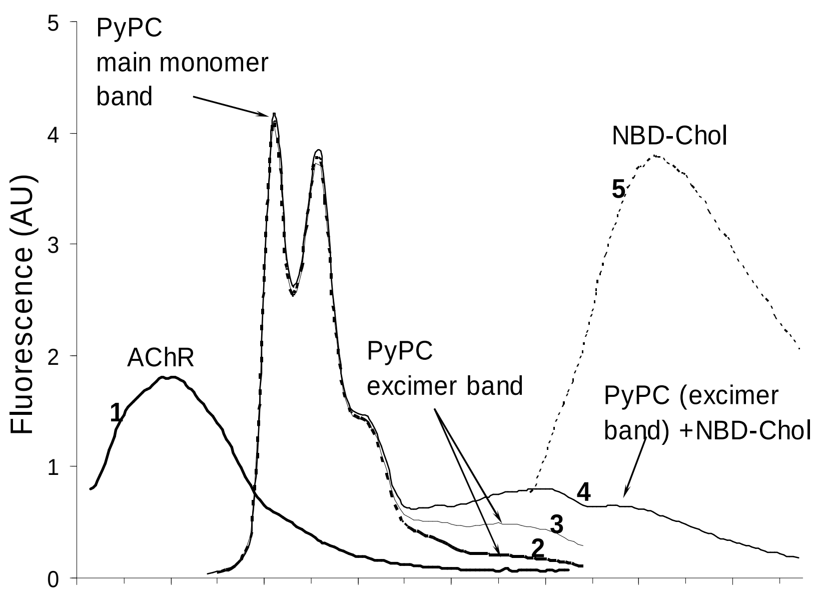

Supplement: Additional File 1 — Reference spectra of AChR, PyPC and NBD-Chol. Fig. 1 and experimental details. [file 1757-5036-1-6-S1.pdf]
