# Supplementary material for: Resolution of complex fluorescence spectra of lipids and nicotinic acetylcholine receptor by multivariate analysis reveals protein-mediated effects on the receptor's immediate lipid microenvironment
Source: PMC Biophys. 2008 Dec 18;1:6. doi: 10.1186/1757-5036-1-6 (PMC2666634; doi:10.1186/1757-5036-1-6)

RMSEP AND EXPLAINED Y-VARIANCE

RMSEP is a measure of the average difference between predicted and measured values at the calibration stage, and can be interpreted as the average modeling error, expressed in the same units as the original values:

$$\text{RMSEP} = \sqrt{\frac{\sum_{i=1}^{n}\left(f_i - \hat{f_i}\right)^2}{n}}$$

where f_i and $\hat{f_i}$ are the known and predicted DOPA molar fraction of the *i*th prediction sample, respectively, and n is the number of predicted samples. The explained y-variance corresponds to the fraction of the total variance which is accounted for by the model and is computed as the complement to the residual variance, divided by the total variance.

Figure 2.

Root Mean Standard Error of Prediction (RMSEP) and percent y-variance explained.

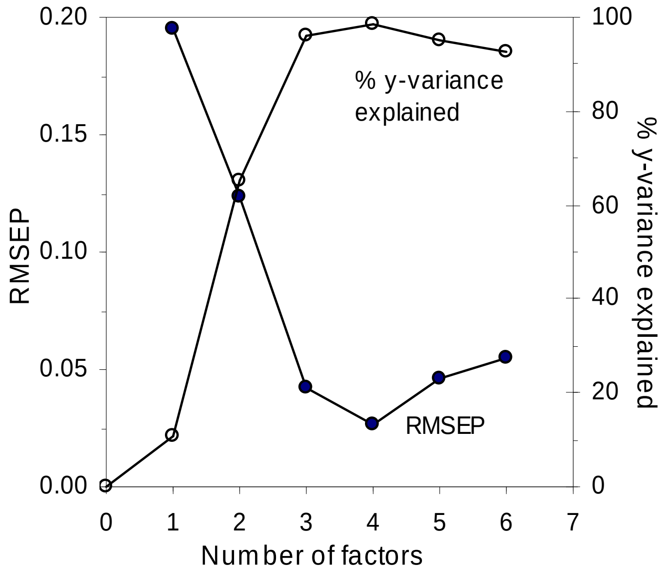

Supplement: Additional File 2 — REMSEP and explained y-variance. Fig. 2 and extra information about these parameters. [file 1757-5036-1-6-S2.pdf]
