# Supplementary material for: Resolution of complex fluorescence spectra of lipids and nicotinic acetylcholine receptor by multivariate analysis reveals protein-mediated effects on the receptor's immediate lipid microenvironment
Source: PMC Biophys. 2008 Dec 18;1:6. doi: 10.1186/1757-5036-1-6 (PMC2666634; doi:10.1186/1757-5036-1-6)

FRET CONTROLS

Figure 3. Fluorescence emission of the AChR and PyPC as a function of temperature in donor-only and acceptor-only samples. A_1 (AChR) and A_2 (PyPC) were computed as the sum of the emission intensities within the interval 304-360 nm and 360-544 nm, respectively (see Fig. 4 in the main text for more details). In order to facilitate comparison of the effect of temperature in both control samples, each area was normalized relative to that with the highest value. Total lipid concentration and lipid to protein molar ratio in the AChR-containing bilayers were 40 μM and 500:1, respectively. Lipid composition: DOPA/DOPC/NBD-Chol (20:40:40). PyPC = 5%. Ex. = 290 nm. Bars in PyPC samples indicate standard deviation of two independent experiments.

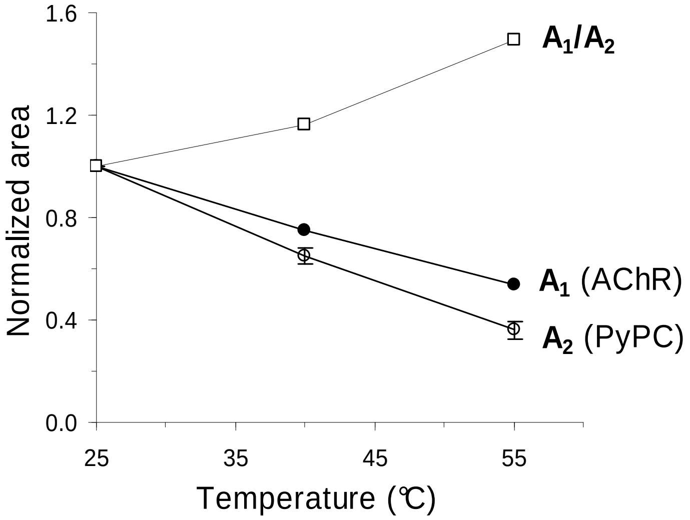

Supplement: Additional File 3 — FRET controls. Fluorescence emission of AChR-only and PyPC-only samples as a function of temperature. Fig. 3 and experimental details. [file 1757-5036-1-6-S3.pdf]
